# Application of lacrimal endoscopy in the diagnosis and treatment of primary canaliculitis: Practical technique and graphic presentation

**DOI:** 10.1097/MD.0000000000016789

**Published:** 2019-08-16

**Authors:** Qingqing Zheng, Ting Shen, Hongbo Luo, Chaoyang Hong, JinJing He, Jingwen Gong, Jin Jiang

**Affiliations:** Department of Ophthalmology, Zhejiang Provincial People's Hospital, People's Hospital of Hangzhou Medical College, Hangzhou, Zhejiang, China.

**Keywords:** canaliculitis, concretions, lacrimal endoscopy, micro drill, surgery technique

## Abstract

To investigate the efficacy of lacrimal endoscopy in the diagnosis and treatment of primary canaliculitis.

This is a retrospective, interventional study. Fifteen patients diagnosed as primary canaliculitis were investigated, who underwent surgery with lacrimal endoscopy from January 2015 to December 2017 at Zhejiang Provincial People's Hospital. Patients were subsequently followed post-operatively for at least 6 months. Pre- and post-operative main measurement included ocular surface symptoms, signs of canaliculitis, intraoperative images, treatment response and complications.

Twelve patients (12 eyes) were enrolled. Endoscopy was successful in revealing the residual concretion and pathological change of lacrimal duct. With its unique direct vision and micro drill, all concretions got removed and lacrimal ducts got patent. On postoperative month 6, all of the patients got cured with no signs of redness, swelling of the punctum and eyelid, epiphora with discharge and pain. None serious complications such as false canalicular passage were observed.

Lacrimal endoscopy is indispensable in diagnosis, treatment and follows up of primary canaliculitis. With less iatrogenic injury, higher resolution rate and direct observation of canalicular mucosa, lacrimal endoscopy should be widely applied in the management of lacrimal diseases.

## 1 Introduction

Characterized by infection of the proximal lacrimal pathway that invariably involves the puncta, canaliculitis is an uncommon infectious disease of lacrimal system. It accounts for <5% of patients with lacrimal disease.^[1,2]^ Patients with canaliculitis usually present with irritation, epiphora, puncta discharge, pouting punctum, and bulbar conjunctival congestion nasally. It usually involves the lower canaliculi and is often unilateral, with a female predominance.^[2,3,4]^ Despite the fact that the underlying cause is not clear, it has been postulated that the presence of a diverticulum or any obstruction of the lacrimal system may predispose to developing canaliculitis as a result of stasis and accumulation of bacteria.^[5,6]^

Although the symptoms and signs of canaliculitis are well known to doctors, it is often overlooked and misdiagnosed as conjunctivitis, chalazion, hordeolum and dacryocystitis. Therefore, delayed treatment and even wrong treatment of canaliculitis are prone to happen in the clinic.

Up to date, several methods have been used to treat canaliculitis. Conservative medical therapy for new onset primary canaliculitis is considered as a first-line treatment.^[7]^ The therapy usually includes manual expression with cotton swab or chalazion forceps, intracanalicular corticosteroid/antibiotic irrigation and microcurettage. Surgical treatments usually contain punctoplasty, canaliculotomy and canalicular curettage combined with lacrimal passages implantation of tube.^[8]^

However, traditional treatment has the following disadvantage. First, we cannot have a clear and definite understanding of the pathological changes in the canaliculus. Second, due to the inevitable Iatrogenic injury to lacrimal mucosa and orbicularis oculi, lacrimal pump dysfunction, canalicular stricture and canalicular scarring often occur, aggravating the severity of primary canaliculitis.^[5,9,10,11]^ Third, we cannot determine whether the concretions are removed thoroughly or not. Finally, there is a high possibility for the appearance of false canalicular passage.

The introduction of lacrimal endoscopy provides an ideal way to solve these thorny problems. Herein we report our experience and skills in the surgical treatment with lacrimal endoscopy in the management of primary canaliculitis.

## 2 Methods

We retrospectively reviewed the medical records of 15 patients (with a total of 15 affected eyes) with canaliculitis who were examined at Zhejiang Provincial People's Hospital from January 2015 to December 2017. This study was approved by the ethics committee of Zhejiang Provincial People's Hospital and was conducted according to the tenets of Declaration of Helsinki. All subjects or their legal guardians gave written informed consent to participate. All treatments were performed by the same surgeons. Clinical data that were obtained included age, gender, involved side and location, presenting symptoms, physical signs, etc.

Inclusion criteria of canaliculitis were:

(1) punctum swelling associated with eyelid thickening and
(2) mucopurulent punctal regurgitation or concretions extruding from the punctum.^[5,6,9,10]^
(3) patients uncured with conservative medical (including repeat compression and intracanalicular antibiotic/ointment infiltration) in the local hospital for at least half a year.

Exclusion criteria were:

(1) patients with secondary canaliculitis, such as those with punctal plug-induced canaliculitis and
(2) patients with dacryocystitis or nasolacrimal obstruction.

**Figure 1:**
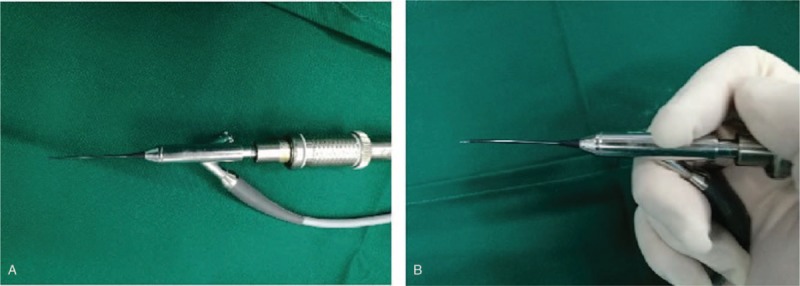
Lacrimal endoscopy working handle. (A) In our surgery, we use a 1.1 mm endoscope with a wash cannula, a channel for the micro optic and a channel for a micro drill. (B) The micro drill is placed into the channel.

After manual compression to remove concretions, all patients underwent surgical intervention with lacrimal endoscopy. The operation was carried out according to the following steps. The patient was placed in the supine position and disinfected routinely. Cotton swab soaked with lidocaine epinephrine solution were insert into the lower nasal meatus of the surgical side. 3 ml of 2% lidocaine infiltration anesthesia was performed to nasolacrimal duct and lacrimal sac. After dilating the punctum, the canaliculus was then probed and irrigated with a 23-gauge blunt lacrimal cannula (attached to a syringe filled with saline). The endoscope (POLYDIAGNOST GmbH) (Fig. 1A) was placed through the upper or lower punctum and navigated through the lacrimal drainage system, allowing direct visualization of the canaliculus, including mucous membrane and potential residual concretions. If concretions were found, flush the concretions to the common canaliculus or lacrimal sac; otherwise, a micro drill (Fig. 1B) was used to crush the concretions before flush in the case where the concretions were too large or closely adhered. Whether or not lacrimal duct intubation was applied would depend on the degree of the lacrimal duct stenosis. After the operation, clan cotton gauze was used to cover the operation eye.

Postoperatively, the patients were prescribed with topical 0.3% tobramycin/0.1% dexamethasone eye drops 6 times per day for 2 weeks, while Tobramycin and Dexamethasone Eye Ointment was applied to the red and swollen area 3 times per day for 1 week. The canaliculus was then probed and irrigated with a 23-gauge blunt lacrimal cannula (attached to a 5 ml syringe filled with Tobramycin/dexamethasone eye drops) to ensure patency for every weak until 1 month after operation. Three months later, the ductule was extracted for the cases with the stent intubated.

The ocular surface symptoms, signs of canaliculitis, treatment response, and any complications were recorded during follow up until 6 months after the operation.

## 3 Results

Among the 15 patients diagnosed with primary canaliculitis enrolled, 3 patients were excluded due to unavailable follow-up information. Finally, 12 patients were included in this study. The demographic data, symptoms, and clinical signs are summarized in Table 1.

**Table 1:**
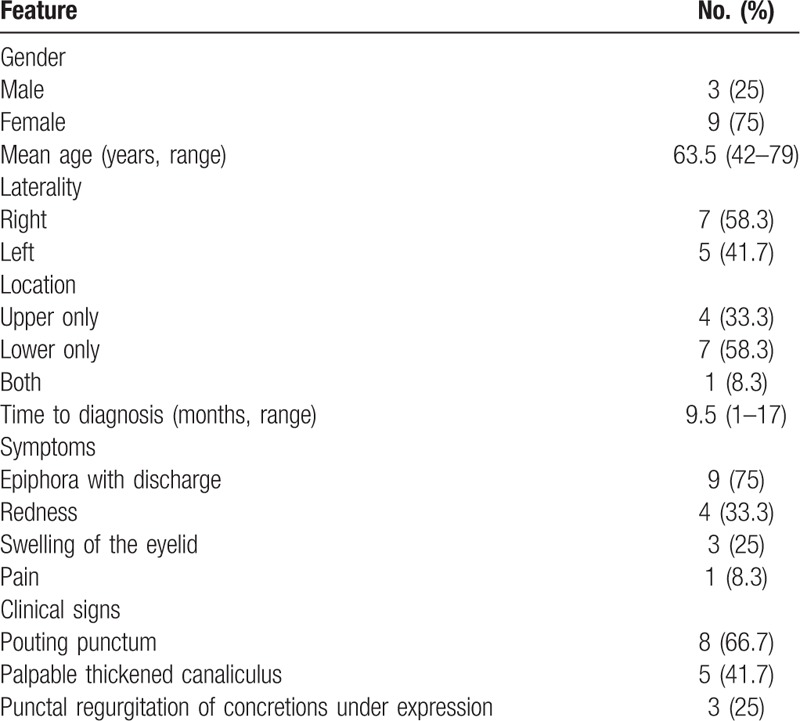
Clinical features of 12 patients with canaliculitis.

**Figure 2:**
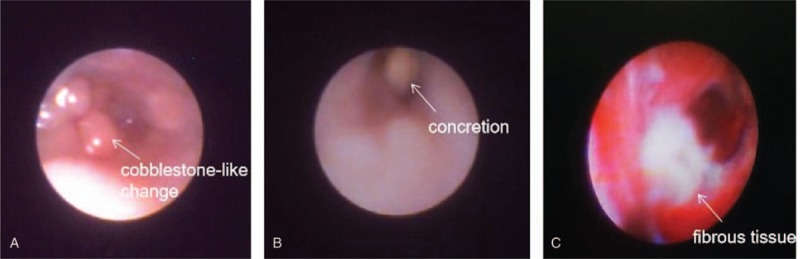
Imaging for primary canaliculitis under lacrimal endoscopy. Lacrimal endoscopy showed obstruction of the canaliculus as a result of cobblestone-like change of the mucous membrane (A), concretion (B), and fibrous tissue (C).

**Figure 3:**
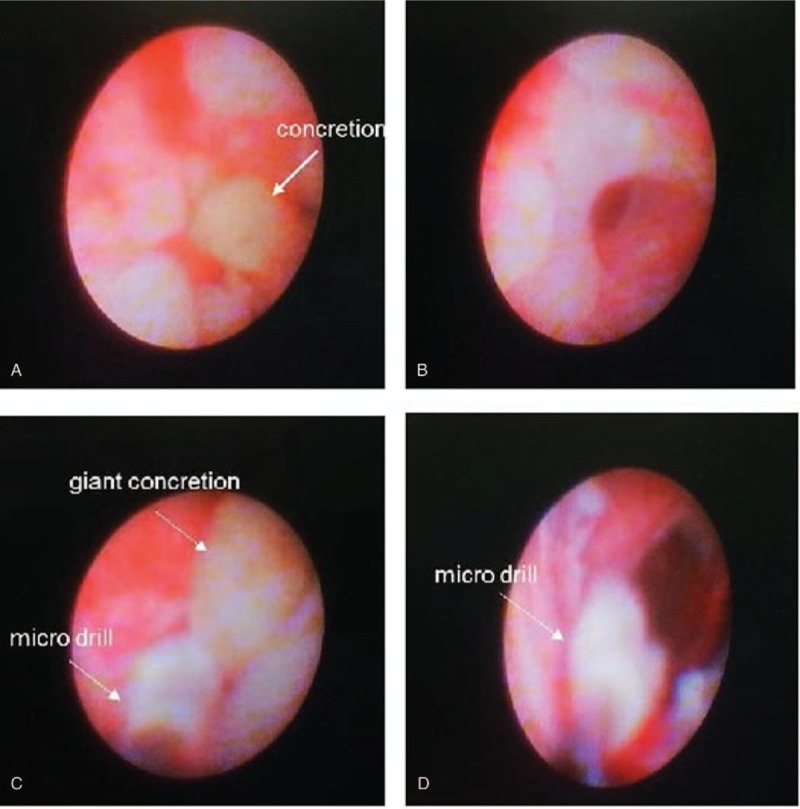
The removal of concretion in the canalicular under lacrimal endoscopy. (A) Lacrimal system endoscopy showed obstruction of the nasolacrimal duct as a result of a concretion. (B) After irrigation, the concretion was gone and nasolacrimal duct became patent. (C) If the concretion was giant, a micro drill was applied to crush the concretion first. (D) And then, it was easy to get rid of the concretion by flush and ensure the patency of duct.

Cobblestone-like change of the mucous membrane (Fig. 2A), mucopurulent discharges, concretions (Fig. 2B), fibrous tissue in the mucosa of canaliculus (Fig. 2C) were observed under lacrimal endoscope in patients. It is easy to observe that concretion obstructed the duct during the operation (Fig. 3A). A flush was required to get rid of them. After irrigation, the concretion was removed and nasolacrimal duct became patent (Fig. 3B). In the case where the concretion was closely adherent to the duct or the concretion was giant, a micro drill was used to divide the concretion into small fragments before irrigation (Fig. 3C and D). Following this, 9 of the 12 patients got lacrimal duct intubated.

**Figure 4:**
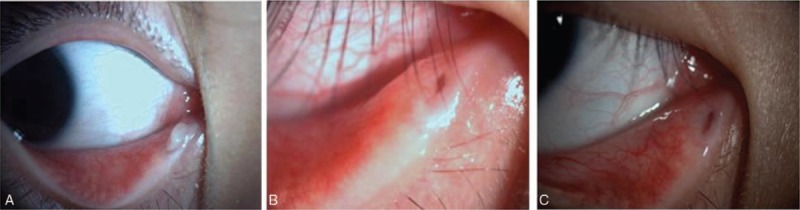
Slit lamp showed the extraocular change before and after the treatment. (A) A large mucopurulent discharge obstructed the lower punctum; the punctum was swollen owing to the canaliculitis. (B) Two weeks after the operation, no discharges were seen near the punctum, with little red and swollen seen around it. (C) It got completely resolved three months later.

In 1 patient, a large mucopurulent discharge and the swollen punctum can be observed when he was enrolled (Fig. 4A). Redness, swelling of the punctum and eyelid, epiphora with discharge and pain were relieved two weeks after the operation (Fig. 4B),and disappeared on postoperative month 3 (Fig. 4C).

By the end of postoperative month 3, all of the patients experienced complete resolution of symptoms and signs except the one with both lacrimal canaliculi involved. The left 1 got cured 1 week later. Irrigation was performed on every follow up, and it demonstrated that there were no obstructions in the lacrimal canaliculus. No discharge, concretions or reflux were found in the process. There were no recurrences in all the patients during the 6 months follow-up. None of these patients were noted to have developed canalicular scarring or narrowing subsequent to the operation.

## 4 Discussion

Lacrimal endoscopy is the first technique that makes it possible to have a direct visualization and precise localization of the lacrimal drainage system and its mucous membranes. It was first introduced by Ebran in 1989.^[12]^ And then, Fein described the endoscopy of lacrimal outflow system from 16 patients in 1992.^[13]^ Endoscopic visualization of the nasolacrimal system was also reported by Singh at the same time. Piffaretti had a key impact on the development of endoscopic instrumentation for diagnostic and therapeutic applications in lacrimal system problems as early as 1993. Following this, specially designed and modified lacrimal endoscopy was developed by Kuchar in 1997.^[14]^ As the picture quality was not satisfactory, a lot of efforts were made to overcome this shortcoming. In recent years, a dedicated lacrimal endoscopy was developed and utilized in clinic. Therefore, not only the normal anatomy visible but also pathological changes such as epithelial changes, polyps, inflammation, lacrimal canaliculus concretions can be achieved in an unprecedented way.

Lacrimal endoscopy is a perfect way to address the defects that the traditional treatments cannot avoid. It has many advantages. First, with the help of lacrimal endoscopy, the diagnosis of canaliculitis becomes easier than any other time in history. The image of punctum, pathologic change of canaliculus and sac (including concretion, diverticulum, false passage, swollen, purulent discharge, foreign body etc) can be precisely observed in great details. Thus, the doctor can determine the state of the canaliculus and possible causes of the disease. Second, under direct visualization the surgeon can remove the canalicular contents and debris perfectly. And it is possible for the surgeon to decide whether it is complete to eliminate the pathological tissue and concretions during the operation. Third, for giant concretion or foreign body, it is feasible that a micro drill can be applied to shatter it into small pieces and then irrigation can be used to eliminate them, keeping unnecessary damages to the minimum. Last but not least, during the post-operative follow-ups, lacrimal endoscopy makes it available to observe the repair of the canalicular mucosa and the stenosis of canaliculi or common canaliculus, and then further timely treatment can be provided to the patient in order to lower recurrence rate. As in our case, lacrimal endoscopy in the surgery and post-operation are widely applied and the therapeutic modalities have an obvious advantage over the previous methods.

However, there exists a learning curve that must be dealt with patiently in this process just like any other new techniques. Anatomical landmarks and disease markers can be easily recognized after repeated practice. After this preliminary stage, surgeons will find it indispensable. The role of endoscopy plays in the diagnosis and treatment of canaliculitis cannot be overestimated.

## Acknowledgments

We thank Wenwei Li for critical reading the manuscript.

## Author contributions

**Conceptualization:** Jin Jiang.

**Data curation:** Qingqing Zheng, Ting Shen, Hongbo Luo, Chaoyang Hong, JinJing He, Jingwen Gong.

**Formal analysis:** Qingqing Zheng, Ting Shen, Hongbo Luo, Chaoyang Hong, JinJing He, Jingwen Gong, Jin Jiang.

**Methodology:** Jin Jiang.

**Writing – original draft:** Qingqing Zheng.

**Writing – review & editing:** Jin Jiang.
